# Measuring the impact of apnea and obesity on circadian activity patterns using functional linear modeling of actigraphy data

**DOI:** 10.1186/1740-3391-9-11

**Published:** 2011-10-13

**Authors:** Jia Wang, Hong Xian, Amy Licis, Elena Deych, Jimin Ding, Jennifer McLeland, Cristina Toedebusch, Tao Li, Stephen Duntley, William Shannon

**Affiliations:** 1 Dept. of Medicine, Washington University School of Medicine, (660 South Euclid Avenue), St. Louis, (63110), USA; 2 St.Louis VA Medical Center, Research Service, (501 North Grand Ave), St. Louis, (63103), USA; 3 Dept. of Neurology, Washington University School of Medicine, (212 N Kingshighway), St. Louis, (63108), USA; 4 Dept. of Mathematics, Washington University, (One Brookings Drive), St. Louis, (63130), USA

**Keywords:** Apnea, BMI, circadian activity patterns, Functional Data Analysis

## Abstract

**Background:**

Actigraphy provides a way to objectively measure activity in human subjects. This paper describes a novel family of statistical methods that can be used to analyze this data in a more comprehensive way.

**Methods:**

A statistical method for testing differences in activity patterns measured by actigraphy across subgroups using functional data analysis is described. For illustration this method is used to statistically assess the impact of apnea-hypopnea index (apnea) and body mass index (BMI) on circadian activity patterns measured using actigraphy in 395 participants from 18 to 80 years old, referred to the Washington University Sleep Medicine Center for general sleep medicine care. Mathematical descriptions of the methods and results from their application to real data are presented.

**Results:**

Activity patterns were recorded by an Actical device (Philips Respironics Inc.) every minute for at least seven days. Functional linear modeling was used to detect the association between circadian activity patterns and apnea and BMI. Results indicate that participants in high apnea group have statistically lower activity during the day, and that BMI in our study population does not significantly impact circadian patterns.

**Conclusions:**

Compared with analysis using summary measures (e.g., average activity over 24 hours, total sleep time), Functional Data Analysis (FDA) is a novel statistical framework that more efficiently analyzes information from actigraphy data. FDA has the potential to reposition the focus of actigraphy data from general sleep assessment to rigorous analyses of circadian activity rhythms.

## 1. Introduction

Activity measured by wrist actigraphy has been shown to be a valid marker of entrained Polysomnography (PSG) sleep phase and is strongly correlated with entrained endogenous circadian phase [1]. Actigraphy data is recorded densely, such as every minute or every 15 seconds, for each patient over multiple days. This data is generally analyzed by reducing the time series activity values to summary statistics such as sleep/wake ratios,[2,3] total sleep time,[2,4] sleep efficiency,[5,6] wake after sleep onset, [2,3,6] ratio of nighttime activity to daytime activity or total activity,[7,8] standard deviation of sleep onset time,[9] and intra-daily variability [10]. More complex modeling of actigraphy includes spectral analysis,[7] cosinor analysis [7] and waveform eduction calculated as an "average waveform" for some period [11].

In this paper we propose a novel statistical framework, Functional Linear Modeling (FLM), a subset of Functional Data Analysis (FDA), for analyzing actigraphy data to extract and analyze circadian activity information through direct analysis of raw activity values [12]. FLM extends standard linear regression to the analysis of functions, which in this case represent circadian activity patterns. FLM is performed by 1) converting a subject's raw actigraphy data to a functional form (i.e., continuous curve over time), and 2) analyzing sets of functions to see if they differ statistically across groups. Our FLM-based analysis shows where and with what level the difference between groups occurs along the time, which provides valuable reference for clinical analysis and treatments, and distinguishes our methods from existing circadian analysis works (see [13] for a review). Moreover, we adopted a non-parametric permutation F test to detect the difference between groups, which makes the results robust to the uncertainty in raw data distribution. Using FLM, we show that the apnea-hypopnea index (apnea) has a statistically significant impact on circadian activity patterns, while body mass index (BMI) in this dataset has little impact.

## 2. Methods

### 2.1 Participants and Measures

Participants were recruited prospectively from the clinic at Washington University in St. Louis Sleep Medicine Center. The sleep center is a multidisciplinary clinic at a tertiary medical facility. Clinic patients with a suspected diagnosis of obstructive sleep apnea (OSA), insomnia, or restless legs syndrome (RLS) were invited to participate. Pregnant women, individuals under age of 18, and patients who report working an evening or overnight shift were excluded from participation due to known biologically different circadian clocks. Clinical covariates such as BMI, co-morbidities, concomitant medications, and presenting sleep complaints were collected. Participants underwent an overnight PSG when clinically indicated. These data were collected in accordance with the standards of the American Academy of Sleep Medicine (AASM) and were reviewed by a board certified sleep physician. PSG data were scored according to the AASM Manual for the Scoring of Sleep and Associated Events. This ongoing study has been approved by the Washington University School of Medicine Institutional Review Board.

Activity was measured using Actical devices (Philips Respironics Inc.) which were positioned on the non-dominant wrist of subjects at the initial sleep center visit and set to measure activity every minute for 7 days. Three hundred and ninety five patients have been recruited, of which 305 have apnea and/or BMI measured. This subgroup comes from a larger NIH funded study currently recruiting a cross section of 750 patients referred to the Washington University Sleep Medicine Center for the purpose of developing and validating functional data analysis methods for actigraphy data (HL092347).

### 2.2. Functional Data Analysis (FDA)

FDA is an emerging field in statistics that extends classical statistical methods for analyzing sets of numbers (scalars for univariate analyses, and vectors for multivariate analyses) to analyzing sets of functions [13][15]. FDA is a subset of the larger field called 'object data analysis' or 'object oriented data analysis' that uses statistical methods to analyze data that are in non-numeric form such as images, graphs (e.g., trees), or functions [14,15]. The goal of object oriented data analysis is to analyze objects in their natural form (e.g., functions, graphs) to extract more information than generally can be extracted when the objects are converted into simpler summary measures (e.g., average activity level, total sleep time) where standard statistical methods can be applied.

#### 2.2.1 Functional smoothing

Functional data analysis (FDA) begins by replacing discrete activity values measured at each time unit (e.g., minute) by a function to model the data and reduce variability. The function represents the expected activity value at each time point measured. Since the actigraphy has equidistant data, to allow flexibility in representing the data as a function, a Fourier expansion model is used, though any smoothing method could be used. Let *y_kj _*be the discrete activity count for patient k at time point *t_kj_*, then the model

(1) $$y_{kj}=Activity_{k}\left(t_{kj}\right)+\varepsilon_{k}\left(t_{kj}\right)$$

represents activity, where *k *= 1, 2,...*,N*,*N *is total number of patients, *j *= 1, 2,...,*T_k_*, *T_k _*is the total number of time points for patient *k*. In our dataset, observation times are minutes from midnight to midnight in 24 hours, so all subjects have the same number of measurements *T_k_*.

We convert the raw actigraphy data to a functional form using a basis function expansion for *Activity_k_*(*t_j_*)

(2) $$\begin{array}{c} Activity_{k}\left(t_{j}\right)=a_{1k}\Phi_{1}\left(t_{j}\right)+a_{2k}\Phi_{2}\left(t_{j}\right)\\ +\cdots +a_{nk}\Phi_{n}\left(t_{j}\right) \end{array}$$

where ${\left\{a_{ik}\right\}}_{i=1}^{n}$ are scalar coefficients for patient k and ${\left\{\Phi_{i}\left(\cdot \right)\right\}}_{i=1}^{n}$ are basis functions. Possible basis functions include polynomials (*f*(*t*) = *a*_1_*t *+ *a*_2_*t*^2 ^+ ... + *a_n_t^n^*), Fourier basis $\begin{array}{l} (f(t)=a_{1}+a_{2}\sin(\omega t)+a_{3}\cos(\omega t)+\\ a_{4}\sin(2\omega t)+a_{5}\cos(2\omega t)+\cdots +a_{n}\varphi_{n}) \end{array}$, splines, and wavelets.

Experimental results (unpublished) show most basis functions work equally well and we have found a Fourier expansion with n = 9 basis functions capture the major trend of activity pattern with reduced noise. Let

$$\begin{array}{c} \Phi_{1}\left(t\right)=1,\;\Phi_{2}\left(t\right)=\cos\left(\omega t\right),\;\Phi_{3}\left(t\right)=\\ \sin\left(\omega t\right),\ldots ,\;\Phi_{8}\left(t\right)=\cos\left(4\omega t\right),\;\Phi_{9}\left(t\right)=\sin\left(4\omega t\right),\;\omega =\frac{2\pi }{T} \end{array}$$

where T is the period, in our case T = 1440 (number of minutes in 24 hours). Equation 1 becomes

$$Activity_{k}\left(t_{j}\right)=\overset{9}{\underset{i=1}{\sum }}a_{ik}\Phi_{i}\left(t_{j}\right)$$

We will use this functional representation for all analyses in this paper.

Smooth coefficients of the expansion ${\left\{a_{ik}\right\}}_{i=i}^{9}$ are estimated by minimizing the unweighted least squares criterion SMSSE [12]:

(3) $$SMSSE\left(y_{k}|a_{k}\right)=\sum_{j=1}^{1440}{\left[y_{jk}-\sum_{i=1}^{9}a_{ik}\Phi_{i}\left(t_{j}\right)\right]}^{2}$$

where *y_k _*= (*y*_1*k*_, *y*_2*k*_,...,*y*_1440*k*_)^'^, *a_k _*= (*a*_1*k*_, *a*_2*k*_,...,*a*_9*k*_)'.

In matrix terms, this criterion becomes:

(4) $$SMSSE\left(y_{k}|a_{k}\right)={\left(y_{k}-\Phi a_{k}\right)}^{\prime }\left(y_{k}-\Phi a_{k}\right).$$

where Φ is a 1440 × 9 matrix with columns for basis functions and rows for basis value at each minute.

Taking the derivative of the criterion *SMSSE*(*y_k_*|*a_k_*) with respect to *a*, gives 2Φ^'^Φ*a_k _*- 2Φ^'^*y_k _*, and setting this equal to 0 and solving for a provides the estimate $\hat{a}$ that minimizes the least square solution,

(5) $${\hat{a}}_{k}={\left(\Phi^{\prime }\Phi \right)}^{-1}\Phi^{\prime }{}_{yk}.$$

Then, the vector $\hat{y}$ of smoothed activity fitted values is

(6) $${\hat{y}}_{k}=\Phi {\hat{a}}_{k}=\Phi {\left(\Phi^{\prime }\Phi \right)}^{-1}\Phi^{\prime }y_{k}$$

The raw data does not need to be normalized since all analyzes are done on the functional form of the data.

To avoid introducing variation between weekday and weekend activity patterns, only data from midnight Monday to midnight Friday was used in this paper, although this simplification is not required for analysis. The five weekdays of actigraphy data were averaged into a single 24 hour profile and a smooth Fourier expansion function was fitted using a 24 hour periodicity and 9 basis functions. This produced a single 24 hour circadian activity pattern for each subject that can be used to estimate patient's activity level at any time point throughout the day. We are developing and preparing to publish functional linear mixed models which will analyze every day's activity data to incorporate day effects, weekday/weekend effects, and pre/post treatment effects which will provide more insight into circadian rhythm patterns and within-subject variability.

**Figure 1:**
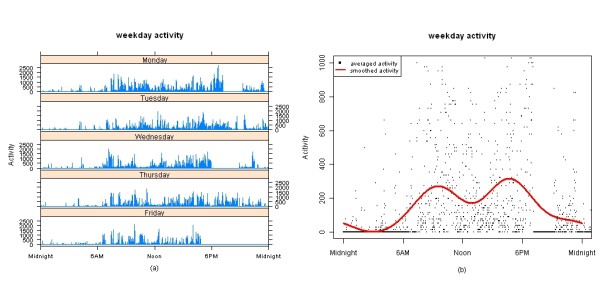
**Data flow for one subject**. Plot (a) shows weekdays ordered Monday through Friday from top to bottom, with the time of day indicated on the X and the height of the spike indicating the raw activity level on the Y axis. The plot (b) shows the activity averaged at each minute over the 5 days (black points) and the Fourier expansion representing this patient's circadian activity pattern (red solid line).

This data smoothing method is illustrated in Figure 1 for a typical subject. Plot (a) shows weekdays ordered Monday through Friday from top to bottom, with the time of day indicated on the X axis running from midnight to midnight, and the height of the spike indicating the raw activity level on the Y axis collected by the actigraphy watch at each minute interval. Plot (b) shows the activity averaged at each minute over the 5 days (black points) and the Fourier expansion representing this patient's circadian activity pattern (red solid line).

#### 2.2.2 Functional Linear Models

Reducing actigraphy data to a summary statistic can mask differences across groups. For example, if one group of patients has high activity in the morning and low activity in the afternoon, and another group has a reversed pattern with the same magnitude of activity, low activity in the morning and high activity in the afternoon, their average activity may be similar, and a significant difference in circadian activity patterns would be missed. FLM avoids masking by extending the linear regression model to the analysis of smooth functions (i.e. circadian activity patterns), and differences such as described in this example become apparent.

**Figure 2:**
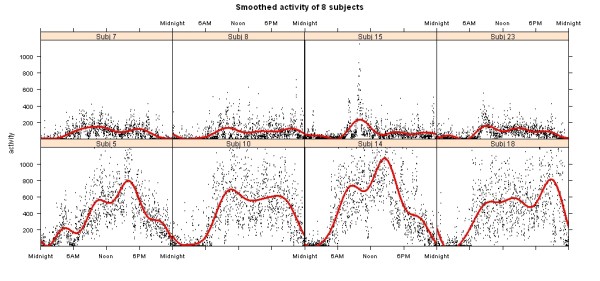
**Smoothed activity of 8 subjects fitted by Fourier expansion and shown in separate plots with time recorded on the X axis, and activity level on the Y axis**. The top 4 plots show the high apnea subjects and the bottom 4 plots show the low apnea subjects.

The conceptual change going from classical linear regression to FLM is that the model regression coefficients, (e.g. *β*_0_, *β*_1_), and error term are functions. To illustrate the use of FLMs for analyzing actigraphy data, four subjects from our database with the highest apnea scores and four subjects with the lowest apnea scores were selected. apnea is a measure of apnea-hypopnea index used routinely in sleep medicine, and measures the severity of sleep apnea with high values indicating more severe disease. In Figure 2, the circadian activity patterns fitted by Fourier expansion for each of the 8 subjects are shown in separate plots with time recorded on the X axis, and activity level on the Y axis. The top 4 plots show the high apnea subjects (severe sleep apnea) and the bottom 4 plots show the low apnea subjects (mild or no sleep apnea). Visually there is a large difference between the circadian patterns in the high and low apnea subjects.

Using this subset of subjects, functional smoothing and linear modeling is illustrated in this section. In the following section the methods are applied to the full dataset.

To test whether high and low apnea patients have different activity levels, standard approaches would reduce each subject's data to an average activity level, and a classical statistical method such as linear regression would test if these values are the same or different. For example, a linear regression model to test if there are differences in average activity between the high apnea (average activity = 78, 76, 80 and 76) and low apnea (average activity = 370, 397, 482 and 421) groups is defined as

(7) $$\text{Activit}{\text{y}}_{\text{k}}=\beta_{0}+\beta_{1}\times AHI+\varepsilon_{k}.$$

where k = 1,2,...,8 are the subjects in Figure 2, apnea is the group membership indicator with apnea = 1 for low apnea subjects, apnea = -1 for high apnea subjects, and *ε_k _*is the error term. The resulting model fit to this data is Activity_k _= 247.9 + 169.9 × apnea, P < 0.001, and R^2 ^= 0.97. The estimated mean activity in the 4 low apnea subjects is 247.9 + 169.9 = 417.8, and in the 4 high apnea subjects is 247.9 - 169.9 = 78. This statistical analysis confirms the clinical belief that apnea impacts activity, and confirms what is seen in Figure 2. However, it does not tell us when during the day activity levels are different.

**Figure 3:**
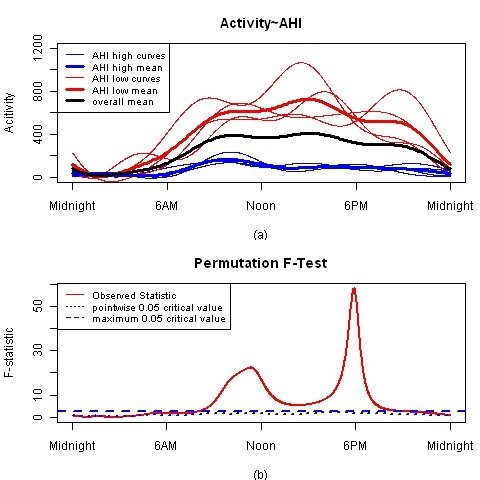
**FLM result for 8 subjects**. Plot (a) shows the 8 individual circadian activity patterns with blue and red line for high and low apnea groups, respectively. The overall mean circadian activity pattern is the solid black line and the mean circadian activity patterns for the high and low apnea groups are thick blue and red line, respectively. Plot (b) shows F-test result the red solid curve represents the observed statistic F(t) at each time point, the blue dashed and dotted lines correspond to a global and point-wise test of significance at significant level α = 0.05, respectively.

Figure 3 illustrates how functional linear modeling is applied to actigraphy data to test for differences between the two apnea groups, and show where during the day those differences occur. Plot (a) shows the 8 individual circadian activity patterns with blue and red line for high and low apnea groups, respectively. The overall mean circadian activity pattern is the solid black line and the mean circadian activity patterns separately for the high and low apnea groups are the thick blue and red line, respectively. Plot (a) shows a clear separation of the mean circadian activity patterns for the two apnea groups and identifies when during daytime those curves differ. In addition, circadian activity behaviors become apparent with this analysis. For example, the maximum activity in the high apnea group (thick blue line) occurs in the morning with a steady decline in activity the remainder of the day, compared to low apnea group (thick red line), the maximum activity occurs at about 3 PM and is stable from about 9 AM to noon and from about 6 PM to 9 PM.

As in the linear regression model described above, we are interested in estimating regression coefficients that will produce the group-specific mean circadian activity patterns, and test if these mean circadian activity patterns are different across groups. This model, for apnea, is defined as [12]:

(8) $$\begin{array}{c} \text{Activit}{\text{y}}_{\text{k}}\left(t\right)=\beta_{0}\left(t\right)+\beta_{1}\left(t\right)\times AHI+\\ \varepsilon_{k}\left(t\right),k=1,2,...N \end{array}$$

where the (t) notation indicates functions over the circadian period for activity (fitted by the Fourier expansion to the actigraphy data for each subject k) Activity_k_(*t*), the mean circadian activity pattern over all subjects *β*_0_(*t*), the functional coefficient indicating how the mean circadian activity patterns changes for low apnea subjects (apnea = 1, *β*_0_(*t*) + *β*_1_(*t*)), or for high apnea subjects (apnea = -1, *β*_0_(*t*) - *β*_1_(*t*)), and *ε_k_*(*t*) is the functional error term. In other words, the low apnea group is predicted to have a mean circadian activity pattern found by adding the two functions *β*_0_(*t*) + *β*_1_(*t*), and the high apnea group is predicted to have a mean circadian activity pattern found by subtracting the two functions *β*_0_(*t*) - *β*_1_(*t*). In Figure 3A* β*_0_(*t*) is the thick black line representing the overall mean, *β*_0_(*t*) + *β*_1_(*t*) is the thick red line for the mean of the low apnea group, and *β*_0_(*t*) - *β*_1_(*t*) is the thick blue line for the mean of the high apnea group.

Equation 8 can be formulated as a matrix analysis problem as described above using a Nx2 design matrix *Z *with rows indicating subjects and columns indicating the mean function (column 1) and effects on the activity due to apnea level *g *(column 2). In standard matrix notation each row is a vector of 1's and -1's indicating if the subject belongs to high apnea (1, -1) and low apnea (1, 1). The two functional linear coefficients are represented in matrix notation as a 'functional vector'

$$\beta \left(t\right)=\left[\begin{array}{c} \beta_{0}\left(t\right)\\ \beta_{1}\left(t\right) \end{array}\right]$$

and the smoothed functional data represented in matrix form by

$$Act\left(t\right)=\left[\begin{array}{c} Activity_{1}\left(t\right)\\ Activity_{2}\left(t\right)\\ \vdots \\ Activity_{N}\left(t\right) \end{array}\right]$$

where each row represents a subject's fitted activity values. Finally, the functional error matrix is defined as *ε*(*t*) = (*ε*_1_(*t*), *ε*_2_(*t*),...,*ε_N_*(*t*))^'^. Equation 8 in matrix notation becomes,

(9) Act(t)=Zβ(t)+ε(t).

The coefficients *β*(*t*) are estimated by minimizing a least squares estimate

(10) $$LMSSE\left(\beta \right)=\overset{N}{\underset{k=1}{\sum }}\int {\left[Act_{k}\left(t\right)-Z_{k}\beta \left(t\right)\right]}^{2}dt$$

where *Z_k _*is the *k^th ^*row of the design matrix *Z*.

After we estimate $\hat{\beta }\left(t\right)$ for *β*(*t*) in function linear regression, we also want to measure the accuracy of our estimation result. We calculate the point-wise 95% confidence limits for these effects using residuals from the model. This formulation is the same as the standard linear model except that instead of numeric coefficients we are now estimating functional coefficients defined over the 24 hour circadian period. A statistical test of the null hypothesis that the circadian activity patterns are the same in both groups is given by the function [12]:

(11) $$F\left(t\right)=\frac{Var\left[{\left(Z\hat{\beta }\right)}_{k}\left(t\right)\right]}{\frac{1}{N}\overset{N}{\underset{k=1}{\sum }}{\left(Act_{k}\left(t\right)-{\left(Z\hat{\beta }\right)}_{k}\left(t\right)\right)}^{2}}$$

where *Z *is the design matrix and $\hat{\beta }$ is a vector of the estimated regression coefficient functions.

Because of the nature of functional statistics, it is difficult to attempt to derive a theoretical null distribution for any given test statistic. Instead, we applied a non-parametric permutation test methodology. If there is no relationship between activity pattern and apnea levels, it should make no difference if we randomly rearrange the apnea group assignment. The advantage of this is that we no longer need to rely on distributional assumptions while the disadvantage is that we cannot test for the significance of an individual covariate among many. The *p *value of the test can then be calculated by counting the proportion of permutation *F *values that are larger than the *F *statistics for the observed pairing. Here we used two different ways to counting the proportion: global test and point-wise test. Global test provides a single number which is the proportion of maximized *F *values from each permutation. Point-wise test provides a curve which is the proportion of all permutation *F *values at each time point.

Plot (b) in Figure 3 provides a display for the statistical significance test for the differences in circadian activity patterns continuously over time. The blue dashed and dotted lines correspond to a global and point-wise test of significance at significant level α = 0.05, respectively, and the red solid curve represents the observed statistic F(t) at each time point. When F(t) is above the blue dashed or dotted line, it is concluded the two apnea groups have significantly different mean circadian activity patterns at those time points. The global critical value (blue dashed line) is preferred since this represents a more conservative test. For these data, the two apnea groups are statistically different in activity from approximately 7 AM - 9 PM.

The statistical and computational details for fitting FLM models are well described elsewhere and are outside the scope of this paper. The reader interested in these details are referred to Ramsay and Silverman [12].

This illustration was meant as an introduction to the methodology only, and not an indicator of a clinical conclusion. In the following section, these methods are applied to the entire 395 subject dataset, and show how apnea and BMI clinically impacts circadian activity patterns.

## 3. Results

### 3.1 Demographic Information

Table 1 shows basic demographic information and sample characteristics. Baseline covariates have been collected from 395 participants (196 females), age ranging from 18 to 80 years old. The average apnea score is 22.1 (standard deviation = 28.1) and average BMI is 34.7 (standard deviation = 8.9). Clinically, BMI > 30 is used to separate subjects into obese and non-obese categories. However, subjects in our database were recruited from a sleep center and had higher BMI than found in the general population, so we cannot generalize our conclusions of the impact of BMI on circadian activity to the entire population.

**Table 1:** Demographic information and sample characteristics

Variable		N (%) Mean ± std (N Total 395)
**Female**		196(49.87%)

**Race**	African-American	134 (35.08%)
	
	Caucasian	237 (62.04%)

**Presenting Symptoms**	Snoring	279 (70.63%)
	
	Gasping	93 (23.54%)
	
	Morning headache	67 (16.96%)
	
	RLS symptoms	26 (6.58%)
	
	PLMS	3 (0.76%)
	
	Witnessed apneas	146 (36.96%)
	
	Insomnia	42 (10.63%)
	
	Excessive day sleepiness	91 (23.04%)
	
	Nonrestorative sleep	9 (2.28%)

**Mallampati score**	Class 4	145 (41.55%)
	
	Class 3	136 (38.97%)
	
	Class 2	53 (15.19%)
	
	Class 1	15 (4.30%)

**Diagnosis Result**	OSA	292 (73.92%)
	
	RLS	5 (1.27%)
	
	Insomnia	8 (2.03%)
	
	Hypersomnia	20 (5.06%)

**BMI > 30**		241 (60.86%)

**BMI**		34.66 ± 8.88 (Median = 34)

**Age(years)**		47.9 ± 14.8

**apnea**		22.11 ± 28.11 (Median = 12.95)

### 3.2 Smoothed Functional Actigraphy Data

**Figure 4:**
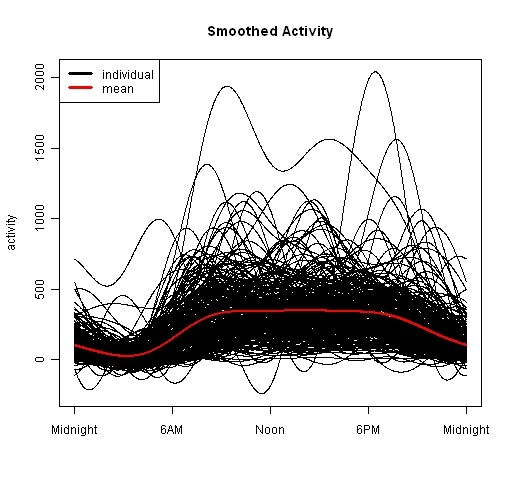
**Smoothed Activity for individuals as black solid curves and overall mean as red curves**.

Raw actigraphy data were read into the R statistical software for analysis using the FDA package and software written by our group to apply FLM methods. Two hundred and eighty nine patients have actigraphy data. Each patient's data from midnight Monday through midnight Friday were averaged and fit by a 9 basis Fourier expansion and their circadian activity patterns plotted in Figure 4. The mean circadian activity pattern across all subjects is shown by the red line. While general structure is visible (e.g., lower activity during sleep hours), the overlap of these curves makes clinically meaningful interpretation difficult.

### 3.3. Functional Liner Model (FLM) Results

We apply FLM to measure the impact of apnea and BMI on subject circadian activity patterns and test the null hypothesis that circadian activity patterns are the same regardless of apnea and BMI values. The alternative hypothesis is that apnea, BMI, and/or their interaction effect activity behavior in a statistically significant way. In addition to the tests of hypotheses, FLM provides a graphical view of the subgroup circadian activity patterns that can aid interpretation of behavioral differences.

To fit these models, each subject is categorized according to their apnea and BMI values by:

$$\begin{array}{c} \text{AHI}=\left\{\begin{array}{cc} \text{1} & \text{if AHI}<\text{Median of AHI}\\ \text{ - 1} & \text{if AHI}\geq \text{Median of AHI} \end{array}\right)\;\;\\ \text{BMI}=\left\{\begin{array}{cc} \text{1} & \text{if BMI}<\text{30}\\ \text{ - 1} & \text{if BMI}\geq \text{30}\text{.} \end{array}\right) \end{array}$$

For the 295 subjects, 235 subjects had data on apnea and actigraphy, 277 subjects had BMI and actigraphy, and 232 subjects had apnea, BMI, and actigraphy. The following analyses are based on these subsets.

We fit the following three functional linear models as defined in Table 2. The first two models measure the univariate impact of apnea (N = 235) and BMI (N = 277) separately, and the third model measures their multivariate impact (N = 232). The models are presented in this order to go from a less to more complicated analysis. Converting apnea and BMI into binary categories was done for simplification but is not necessary for functional linear modeling, and continuous apnea, BMI, or other covariates could be used. At the end, we show how BMI can be analyzed by FLM as a continuous variable.

**Table 2:** Three Functional Linear Models

Model 1	apnea Main Effect Only	Activity*_k_*(*t*) = *β*_0_(*t*) + *β_AHI_*(t) × AHI_k _+ *ε_k_*(*t*)
Model 2	BMI Main Effect Only	Activity*_k_*(*t*) = *β*_0_(*t*) + *β_BMI_*(t) × BMI_k _+ *ε_k_*(*t*)

Model 3	apnea+BMI+interaction	Activity*_k_*(*t*) = *β*_0_(*t*) + *β_AHI_*(t) × AHI_k _+ *β_BMI_*(t) × BMI_k _+*β_AHI _*× _*BMI *_(t) × AHI_k _× BMI*_k _*+ *ε_k_*(*t*)

#### 3.3.1 Apnea Main Effect Models

The impact of apnea as a main effect on circadian activity patterns was tested with Model 1, Table 2. The null hypothesis is that the circadian actigraphy patterns are the same in the two apnea groups. Of the 235 subjects in this analysis, 118 have apnea less than the median apnea = 10.8, and 117 patients have apnea larger than or equal to 10.8.

**Figure 5:**
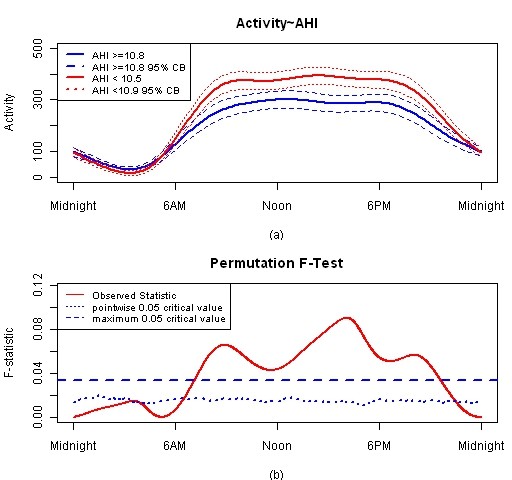
**FLM result for apnea main effect model**. Plot (a) is estimated activity patterns for two apnea groups and 95% confidence band. Plot (b) is F-test result for this model.

Figure 5 presents the estimated group means with 95% confidence bands in plot (a). The low apnea group indicating less disease severity (red solid line) has higher activity during the day compared to the high apnea group (blue solid line). The confidence bands around the two group mean curves do not overlap during the day suggesting the variability in the group circadian activity patterns do not cross. The F-test in the plot (b) indicates when these curves are statistically different during the day. The F-test result shows that the two apnea groups are significantly different from about 7 AM to 9 PM.

#### 3.3.2. BMI main effect

**Figure 6:**
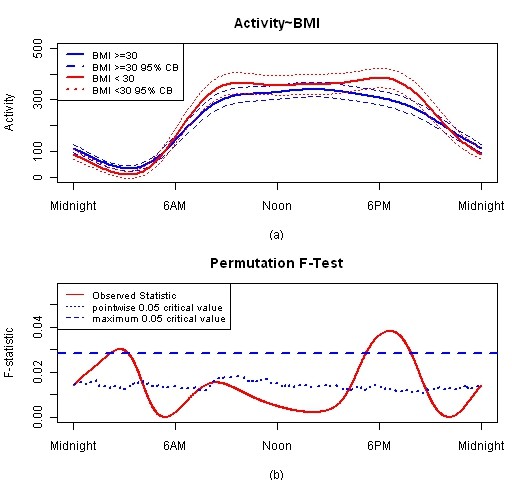
**FLM result for BMI main effect model**. Plot (a) is estimated activity patterns for two BMI groups and 95% confidence band. Plot (b) is F-test result for this model.

Next, the impact of BMI as a main effect on circadian activity patterns was measured using Model 2, Table 2. The null hypothesis is that the circadian activity patterns are the same in non-obese (BMI < 30) and obese (BMI > = 30) groups. 182 patients are classified as obese and 95 as non-obese. Figure 6 presents estimated group means with 95% confidence band and F-test result. The high BMI group (blue solid line) has higher activity during night and lower activity during daytime, but activity patterns for the two groups are only significantly different around 3 AM and 6 PM.

We emphasize that the population of participants in this study had a higher overall BMI compared to the general population which may explain why the expected difference in circadian activity patterns across these groups was not observed.

#### 3.3.3 Apnea and BMI effect, with interaction

Model 3, Table 2 was used to measure the impact of apnea, BMI, and the apnea × BMI interaction term on circadian activity patterns. The null hypothesis is that the circadian activity patterns measured are the same in the four apnea × BMI groups versus the alternative that apnea and/or BMI and/or their interaction impacts circadian activity patterns in a statistically significant way. Table 3 shows the sample sizes for each group.

**Table 3:** Sample size for apnea, BMI mode

	apnea Low (< 10.75)	apnea High (> = 10.75)	Total
**BMI > = 30**	61	94	155

**BMI < 30**	55	22	77

**Total**	116	116	232

This interaction model has four functional coefficients $\left({\hat{\beta }}_{o}\left(t\right),{\hat{\beta }}_{AHI}\text{(t),}{\hat{\beta }}_{BMI}\text{(t)},{\hat{\beta }}_{AHI\times BMI}\text{(t)}\right)$ which in combination define the four clinical groups (e.g., low BMI and low apnea; low BMI and high apnea, etc). The four subgroups' circadian activity can be estimated by adding or subtracting the functional coefficients as shown in Table 4.

**Table 4:** Four group circadian activity result

apnea	BMI	Group Mean
Low	Low	${\hat{\beta }}_{o}\left(t\right)+{\hat{\beta }}_{AHI}\text{(t)}+{\hat{\beta }}_{BMI}\text{(t)}+{\hat{\beta }}_{AHI\times BMI}\text{(t)}$

Low	High	${\hat{\beta }}_{o}\left(t\right)+{\hat{\beta }}_{AHI}\text{(t)}-{\hat{\beta }}_{BMI}\text{(t)}-{\hat{\beta }}_{AHI\times BMI}\text{(t)}$

High	Low	${\hat{\beta }}_{o}\left(t\right)-{\hat{\beta }}_{AHI}\text{(t)}+{\hat{\beta }}_{BMI}\text{(t)}-{\hat{\beta }}_{AHI\times BMI}\text{(t)}$

High	High	${\hat{\beta }}_{o}\left(t\right)-{\hat{\beta }}_{AHI}\text{(t)}-{\hat{\beta }}_{BMI}\text{(t)}+{\hat{\beta }}_{AHI\times BMI}\text{(t)}$

**Figure 7:**
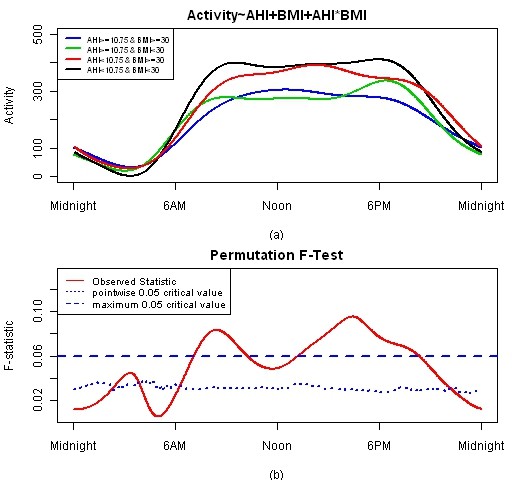
**FLM result for apnea and BMI model**. Plot (a) is estimated activity patterns for the four groups and 95% confidence band. Plot (b) is F-test result for this model.

When a subject's apnea or BMI is low, the functional coefficient for that factor is added to the mean activity pattern. When a subject's apnea or BMI is high, the functional coefficient for that factor is subtracted from the mean activity pattern. The interaction coefficient is added when apnea and BMI are concordant (high/high or low/low) and subtracted when apnea and BMI are discordant (low/high, high/low). Figure 7 shows the activity curves for each of the four groups defined according to their apnea/BMI status. The F-test shows a significant difference among these four group activity patterns between about 7 AM to 11 AM and 12:30 PM to 8 PM.

It is an established statistical practice in a linear regression model to test the main effects of two covariates and the effect of the interaction of the two covariates. We extended this method to the functional linear model. The comparisons of all 4 groups in this section are actually the evaluation of the combination of the main and interaction effects which should be consistent with a 2-way ANOVA.

#### 3.3.4 BMI as a Continuous Variable

**Figure 8:**
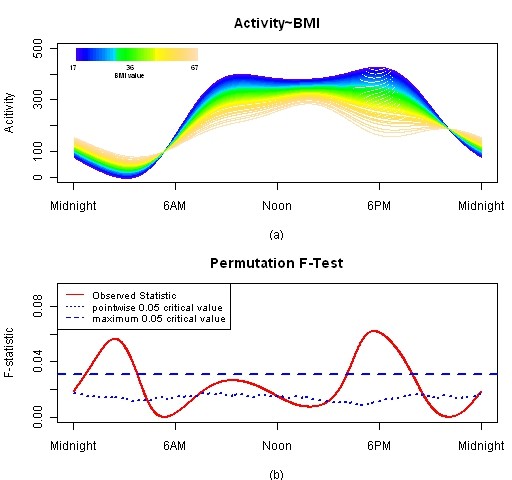
**FLM result for BMI model treating BMI as continuous**. Plot (a) is estimated activity patters for BMI groups. Plot (b) is F-test result for this model.

As noted above, BMI showed little impact on circadian activity patterns which does not correspond to general clinical belief. This is most likely explained by the fact that our subject population has high BMI relative to the general population, so the distinction between obese and non-obese was less pronounced. In this section, we fit a functional linear model treating BMI as a continuous variable. BMI ranges from 17 to 67 in this dataset. Figure 8 presents estimated means and F-test result. In this plot, each color represents one BMI group. The largest BMI group has higher activity during night and lower activity during daytime. BMI impact is significant around 1 AM to 4 AM and 4 PM to 8 PM. It is noted that the significantly different time periods are longer than those obtained from categorized BMI effect model.

## 4. Discussion

Traditionally, actigraphy data is transformed into summary numbers, such as total sleep time, sleep efficiency, wake after sleep onset, and other measurements. These transformations allow data analysts to test hypothesis using simple classical statistical methods. However, large amount of information can be lost and problems of masking circadian patterns may arise.

The merit of functional linear modeling relies in determining when along the 24-hour scale groups differ. Results from parameter tests in a cosinor approach would provide information as to differences in harmonic content between groups. Another advantage of the functional linear modeling approach is exemplified in Figure 8, where BMI is used as a variable instead of comparing groups with higher versus lower BMI values.

In this paper we have presented a novel approach for analyzing the full actigraphy data which we believe avoids significant information loss and masking effect. Representing actigraphy data as smooth continuous functions, and applying Functional Linear Modeling methods allowed us to directly compare and test differences of circadian activity patterns across apnea and BMI subgroups. Other Functional Data Analysis methods using principal components analysis ([15]; Zeitzer, et al. 'Phenotyping apathy in individuals with Alzheimer's using functional principal component analysis', Revised and Resubmitted) for identifying sources of variability within circadian activity patterns across subgroups, and mixed effect models (Ding, et al., 'Functional Linear Mixed Effects Model for Actigraphy Data', In Preparation) for incorporating additional sources of within subject variability are currently being developed in our lab and applied to this type of data. Functional linear mixed models are also being developed in our lab which will allow within-subject variability such as day-to-day or pre-treatment to post-treatment differences in activity to be analyzed.

## Competing interests

The authors declare that they have no competing interests.

## Authors' contributions

JW and HX carried out statistical analysis, contributed to development of methodology and wrote sections of the manuscript. AL provided clinical input and oversight. ED developed the clinical database, contributed to statistical programming and reviewed the manuscript. JD developed theoretical mathematical basis for the analysis and wrote section of the manuscript. JM and CT acted as clinical coordinators, entered the data, wrote sections and critically reviewed the manuscript. TL provided programming and mathematical support and critically reviewed the manuscript. SD is co-PI on the project, oversaw all clinical aspects of the project, provided clinical theoretical perspectives and wrote sections of the manuscript. WS was the PI on the project, developed statistical methodology, oversaw the work of statisticians and programmers, wrote sections of the manuscript and critically reviewed all its contents. All authors have read and approved the final manuscript.
